# ANALYSIS OF THE CLINICAL CHARACTERISTICS OF PERIANAL FISTULISING CROHN’S DISEASE IN A SINGLE CENTER

**DOI:** 10.1590/0102-672020180001e1420

**Published:** 2019-02-07

**Authors:** Min-Min XU, Ping ZHU, Hao WANG, Bo-Lin YANG, Hong-Jin CHEN, Li ZENG

**Affiliations:** 1 Department of Colorectal Surgery, Affiliated Hospital; 2 The First Clinical Medical College, Nanjing University of Chinese Medicine, Nanjing, Jiangsu, China.

**Keywords:** Fistul, Crohn diseas, Population characteristic, Doença de Croh, Fístul, Características da populaçã

## Abstract

**Background:**

Clinical characteristics are keys to improve identification and treatment of Crohn´s disease (CD) so that large sample analysis is of great value.

**Aim:**

To explore the clinical characteristics of perianal fistulising CD.

**Methods:**

Analysis of 139 cases focused on their clinical data.

**Results:**

The proportion of males and females is 3.3:1; the mean age is 28.2 years; 47.5% of patients had anal fistula before CD diagnosis. Patients with prior perianal surgery and medication accounted for 64.7% and 74.1% respectively. The L3 type of lesion was present in 49.6% and the B1 and B2 types for 51.8% and 48.2% respectively; complex anal fistula was diagnosed in 90.6%. Symptoms of diarrhea were found in 46% and perianal lesions alone in 29.5% of patients. Abnormal BMI values was present in 44.6%; active CD activity index in 64.7%; and 94.2% had active perianal disease activity index. A proportion of patients manifest abnormal C-reactive protein, erythrocyte sedimentation rate, platelet, hemoglobin and albumin.

**Conclusion:**

We suggest that patients with anal fistula associated to these clinical features should alert the medical team to the possibility of CD, which should be further investigated through endoscopy and imaging examination of alimentary tract to avoid the damage of anal function by routine anal fistula surgery.

## RESUMO

**Racional:**

As características clínicas são fundamentais para melhorar a identificação e o tratamento da doença de Crohn (DC), de modo que a análise da amostra seja de grande valor.

**Objetivo:**

Explorar as características clínicas da DC fistulizante perianal.

**Métodos:**

Análise de 139 casos focados em seus dados clínicos.

**Resultados:**

A proporção de homens e mulheres foi de 3,3: 1; a média de idade de 28,2 anos; 47,5% dos pacientes tiveram fístula anal antes do diagnóstico de DC. Pacientes com cirurgia perianal prévia e medicação representaram 64,7% e 74,1%, respectivamente. O tipo de lesão L3 estava presente em 49,6% e os tipos B1 e B2, em 51,8% e 48,2%, respectivamente; fístula anal complexa foi diagnosticada em 90,6%. Sintomas de diarréia foram encontrados em 46% e lesões perianais isoladas em 29,5% dos pacientes. Valores anormais de IMC estavam presentes em 44,6%; índice de atividade DC ativa em 64,7%; e 94,2% tinham índice de atividade de doença perianal ativo. Proporção significativa de pacientes tinha proteína-C reativa, taxa de sedimenta do eritrócito, plaquetas hemoglobina e albumina anormais.

**Conclusão:**

Sugere-se que pacientes com fístula anal associada às essas características clínicas alertem a equipe médica para a possibilidade de DC, que deve ser investigada por endoscopia e exame de imagem do trato digestivo para evitar dano na função anal pela operação que rotineiramente é realizada no tratamento da fístula anal.

## INTRODUCTION

Anal fistula is the most common perianal lesion of Crohn’s disease (CD). Its incidence has been reported approximately 35%^12^, and 5% of patients first manifest anal fistula^3^. Clinical characteristics are keys to improve identification and treatment, so that large sample analysis is of great value. 

The objective of this study was to explore the clinical features of anal fistula in CD to better indicate the appropriate treatment

## METHOD

This study was approved by the Ethics Committee of the institution under the number 2015NL-126-03

### Research subjects and observation indexes

Cases of 139 perianal fistulising CD patients, who were first admitted to the Affiliated Hospital of Nanjing University of Chinese Medicine from January 2010 to January 2017, were registered to collect their gender, age, CD course, anal fistula course, body mass index (BMI), Montreal classification, anal fistula type, external opening number, concomitant perianal lesions, clinical symptoms, abdominal surgery history, perianal surgery history, medication history, CD activity index (CDAI), perianal disease activity index (PDAI) and laboratory analyses, as C-reactive protein (CRP), erythrocyte sedimentation rate (ESR), leukocyte (WBC), platelet (PLT), hemoglobin (HB), albumin (Alb).

### Criteria of clinical assessment and classification

The nutritional status was evaluated by BMI. The Montreal Phenotypic Classification^14^, proposed by the 2005 World Gastroenterology Working Group, was used to classify CD. It´s activity index was evaluated by Best’s CDAI^2^ calculation. The activity of anal fistula in CD was evaluated by PDAI^7^. Anal fistula was divided into simple and complex types^1^ according to the 2003 American Gastroenterological Association Statement on Perianal CD.

## RESULTS

Table 1 shows basic statistics of the 139 cases, including gender, CD course, anal fistula course, surgery history and medication history. The proportion of male to female is about 3.3:1. The CD courses range from 0 month to 12 years, with an average of 28±38 months. Anal fistula courses range from 0 month to 10 years, with an average of 25±29 months. Twenty-seven patients had abdominal surgery history, accounting for 19.4%. Ninety had perianal surgery history, accounting for 64.7%. Perianal abscess surgeries were related in 40.3% of patients, holding the largest proportion, followed by anal fistula surgery in 27.3%. Medication history accounted in 74.1% of patients and most of them (44.6%) used 5-aminosalicylic (5-ASA). 

**TABLE 1:** The basic clinical features

Item		n (%)
Gender	male	107 (77.0)
	female	32 (23.0)
CD course	<1year	79 (56.8)
	1~10year	54 (38.8)
	>10 year	6 (4.3)
Anal fistula course	<1 year	74 (53.2)
	1~10 year	60 (44.6)
	>10 year	5 (3.6)
Relationship between the two courses	Anal fistula appears first Simultaneously CD diagnosed first	66 (47.5%) 27 (19.4%) 46 (33.1%)
Number of previous abdominal surgeries	1	23 (16.5)
	>1	4 (2.9)
Perianal surgeries history	Anal fistula surgery	38 (27.3)
	Perianal abscess surgery	56 (40.3)
	Hemorrhoids surgery	6 (4.3)
	Anal fissure surgery	2 (1.4)
	Else♦	1 (0.7)
Number of previous perianal surgeries	1	58 (41.7)
	>1	32 (23.0)
Medication history	Yes 5-ASA	103 (74.1) 62 (44.6)
	SASP	15 (10.8)
	Immunosuppressor	24 (17.3)
	Corticosteroid	31 (22.3)
	Biologics	22 (15.8)
	Antibiotic	16 (11.5)
	Enteral nutrition	28 (20.1)
	Else*	23 (16.5)

♦ =resection of perianal mass; * =treatment of Chinese herbal pieces, extract of Chinese herbal pieces, transplantation of fecal bacteria, anti-tuberculosis, antiviral treatment and so on.

The Montreal phenotype are shown in Table 2. The age ranged from 13 to 59 years (28.2±8.48), and 85.6% of them were of the type A2. Type L3 had the highest proportion up to 49.6%. The types B1 and B2 were common, accounting for 51.8% and 48.2% respectively. In this data, the anorectal stenosis was included in type B2 and the penetration of anal fistula was excluded from type B3.

**TABLE 2:** The Montreal phenotypes

Item		n (%)
Age (A)	A1 (≤16)	6 (4.3)
	A2 (17~40)	119 (85.6)
	A3 (>40)	14 (10.0)
Location (L)	L1 (ileum)	33 (23.7)
	L2 (colon)	37 (26.6)
	L3 (ileocolon)	69 (49.6)
	L4 (upper gastrointestinal)	5 (3.6)
Behavior (B)	B1 (nostricturing, no penetrating)	72 (51.8)
	B2 (stricturing)	67 (48.2)
	B3 (penetrating)	10 (8.6)

Anal fistula type, external opening number, and other concomitant perianal diseases are shown in Table 3. The incidence of complex anal fistula is relatively higher, up to 90.6%. The incidences of anorectal stenosis and verrucous skin were 33.1% and 30.9%, higher than those of other concomitant anal diseases.

**TABLE 3:** Anal fistula type, external opening number, and other concomitant perianal diseases

Item		n (%)
Anal fistula type	Simple anal fistula	13 (9.4)
	Complex anal fistula	126 (90.6)
External opening number	1	48 (34.5)
	2	44 (31.7)
	≥3	47 (33.8)
Concomitant perianal diseases	Anorectal stenosis	46 (33.1)
	Fissure	6 (4.3)
	Abscess	33 (23.7)
	Rectovaginal fistula	7 (5.0)
	Verrucous skin	43 (30.9)

The clinical symptoms are shown in Table 4. Diarrhea was the most common symptom, accounting for 46% of the 139 patients. Meanwhile, 29.5% merely have perianal symptoms.

**TABLE 4:** The clinical symptoms

Item	n (%)
Abdominal pain	30 (23.0)
Diarrhea	64 (46.0)
Fever	27 (19.4)
Loss of weight	37 (26.6)
Anaemia	21 (15.1)
Hematochezia	23 (16.5)
Perianal symptoms only	41 (29.5)

The BMI, CDAI and PDAI values are shown in Table 5. Abnormal BMI values were found in 44.6% and 35.3% had CDAI ratings classified as “Very Well”, i.e. in remission, while 38.1% have ratings of “Fair to Good”. According to their PDAI scores, 94.2% of patients were in active stage of perianal diseases.

**TABLE 5:** The BMI, CDAI and PDAI values

Item		n (%)
BMI	<18.5 kg/m2	62 (44.6)
	≥18.5 kg/m2	77 (55.4)
CDAI	Very well (<150 points)	49 (35.3)
	Fair to good (150~220 points)	53 (38.1)
	Poor (221>450 points)	37 (26.6)
	Very poor (>450 points)	0 (0)
PDAI	Remission stage (≤4 points)	8 (5.6)
	Active stage (>4 points)	131 (94.2)

The laboratory analyses are shown in Table 6. CRP increasing was present in 38.1% of patients; 53.2% for ESR accelerating, 38.8% for HB decreasing, 36.0% for PLT increasing, 23.7% for Alb decreasing.

**TABLE 6:** The common laboratory indexes

Item		n (%)
CRP (mg/l)	≥8	53 (38.1)
	<8	86 (61.9)
ESR^a^ (mm/h)	Above normal	74 (53.2)
	Normal	65 (46.8)
HB^b^ (g/l)	Below normal	54 (38.8)
	Normal	85 (61.2)
PLT (109/l)	<100	1 (0.7)
	≥300	50 (36.0)
	100~300	88 (63.3)
WBC (109/l)	<10	13 (9.4)
	4~10	115 (82.7)
	>4	11 (7.9)
Alb (g/L)	35~50	103 (74.1)
	<35 >50	33 (23.7) 3 (2.2)

^a^=The normal value for male is 0~15 mm/h, and 0~20mm/h for female; ^b^=The normal value for male is 120~160 g/l and 110~150 g/l for female

## DISCUSSION

Studies in developed countries suggest that the incidence of CD is bimodal distribution, reaching the first peak at the age of 20-39 and second and lower peak at the age of 60-79^10^. But according to epidemiological studies in China, the second peak is not significant^9^. In our study, the mean age of patients was 28.2 years, and 85.6% were type A2, which is close to the study in Southern China by Song XM et al.^15^, reporting it in 70.7% among 205 patients. In western countries, the distribution of lesion locations is even, but in China the type L3 is most common, with a ratio of 61-71%^18^ ^,^ ^19^. In our study, type L3 accounted for 49.6% of cases, slightly lower than other China reports, but still significantly higher than type L1 and type L2. As for disease behaviors, Ng et al^11^reported that the ratios of B1, B2 and B3 of CD patients in Hong Kong were 65.2%, 25.1% and 16.1%, respectively. Zhao Jet al^19^ reported that the B1, B2 and B3 ratios were 44%, 29%, and 24%, respectively. In our study, the ratio of B1 was 51.8%, which is consistent with other China studies, but the ratio of B2 of 48.2% was significantly higher than other China studies. One of the possible reasons is that 33.1% of perianal fistulising CD patients with anorectal stenosis are classified as L2.

In most western developed countries, the number of female CD patients is more than male^9^, and the ratio of male to female is 1:1.46. On the contrary, in Asia the ratio is near 1:1, and, in many cases, male are far more^8^ ^,^ ^11^ ^,^ ^15^. In our study, the male and female ratio was near 3.3:1, which is consistent with other Asian reports. Perianal surgery history was present in 64.7% mostly for perianal abscess and anal fistula, and 23% had more than one surgery. Medication use of 5-aminosalicylic derivatives were related by 44.6% of patients, while the 2016 ECCO Consensus^5,6^ denied the clinical benefits of 5-aminosalicylic derivatives for CD patients, and recommended IFX, immunosuppressive drugs and antibiotics for perianal fistulising CD.

The prevalence of anal fistula varies according to disease location. Perianal fistulae were noted in 12% with isolated ileal disease, 15% with ileocolonic disease, 41% with colonic disease and rectal sparing, and 92 % with colonic disease involving the rectum^5^. In our study, anal fistulas are usually accompanied by anorecal stenosis and verrucous skin, suggesting that proctitis exists. Due to the progress in itself CD can lead to internal and external sphincter and perineal damage, and proctitis leads to reduced rectal compliance. Even moderate level of sphincter function decline may eventually lead to incontinence because of obstruction of water absorption in the colon and decreased rectal capacity and compliance^16^. Therefore, patients of perianal fistulising CD with proctitis should accept abscess drainage or non-cutting seton, rather than definitive surgery to promote fistula closure^4^.

The ECCO Consensus^5^ suggested that perianal diseases may occur before intestinal symptoms or simultaneously to it. Schwartz D A et al^13^ found that only 5% of CD patients first manifest anal fistula without any intestinal inflammation. In this study, 47.5% of patients had anal fistula before the occurrence of intestinal inflammation, and 29.5% presented perianal lesions only. These percentages, which are much higher than previous report^13^, suggest that isolated anal fistula without intestinal inflammation could not rule out the possibility of CD. “Very well” or “Fair to Good” stages of CDAI were present in 73.4%, but 94.2% of them were in active stage of PDAI, i.e. their intestinal inflammations were controlled better than their perianal diseases. The possible reason is that most of our patients had received basic treatment to control intestinal inflammation before they came to our hospital.

Malnutrition is one of the most common symptoms of IBD, and its incidence is up to 85%. Protein energy malnutrition is the most common type, characterized by emaciation and weight loss. Its incidence is associated with reduced intake of food in fear of bowel symptoms triggered by eating, and loss of nutrients and nutritional in taking disorders caused by intestinal lesions. Some of our patients showed low BMI, low Alb, low HB and weight loss. These abnormalities remind us to assess and correct the nutritional status of patients. Endoscopic mucosal healing is the goal of CD treatment; previous study^17^ have shown that CD simplified endoscopic score (SES-CD) is correlated with the commonly used clinical indexes of inflammation, e.g. CRP, ESR and PLT, but the accuracy is poor. The method of calprotectin detection has higher accuracy to detect endoscopic activity, compared to other clinical and laboratory indexes. In this study, patients with high ESR, CRP and PLT account for 53.2%, 38.1% and 36%, respectively, indicated activity of intestinal inflammation. Our center has not yet carried out faecal calpain detection, so, there was no relevant laboratory data.

## CONCLUSION

We suggest that patients with anal fistula associated to these clinical features should alert the medical team to the possibility of CD, which should be further investigated through endoscopy and imaging examination of alimentary tract to avoid the damage of anal function by routine anal fistula surgery.
